# Molecular characterisation of KRAS mutations in non-small cell lung cancer across all stages

**DOI:** 10.3332/ecancer.2025.1914

**Published:** 2025-05-27

**Authors:** Carla Climent, Sandra Soriano, Natalia Lopez, Julia Giner, Mari Carmen Blazquez, Ruben Carrera, Marina Sierra, Pablo Cobo, Monica Fragio, Mireia Busquets, Ona Cano I Cano, Alicia Carrasco, Miguel Ángel Seguí, Laia Vila

**Affiliations:** 1 Department of Medical Oncology, Consorcio Hospital Universitario Parc Tauli, Sabadell 08208, Spain; 2 Department of Pathological Anatomy, Consorcio Hospital Universitario Parc Tauli, Sabadell 08208, Spain

**Keywords:** non-small cell lung cancer, KRAS mutation, co-mutations, PD-L1

## Abstract

**Introduction:**

Kirsten rat sarcoma virus (KRAS) mutations (KRASms) are detected in approximately 25% of non-small cell lung cancer (NSCLC) patients with adenocarcinoma. Next-generation sequencing (NGS) has enabled the identification of diverse KRASm subtypes with distinct prognoses, co-mutation patterns and clinical characteristics. This study aimed to investigate the clinical and pathological characteristics of KRASm patients across all stages of NSCLC.

**Methods:**

We analysed NSCLC patients from 2019 to 2021 using the Illumina Focus 52-gene targeted NGS panel, which detects DNA and RNA alterations. PD-L1 expression was assessed using the SP263 antibody. We examined the clinical and pathological characteristics of KRASm patients, including KRASm subtypes and co-mutations.

**Results:**

Of the 123 patients, 62 (50.4%) had KRASm, with a median age of 67 years (range 49–92). Of these, 79% were male, 87.1% had adenocarcinomas and only 8.1% were non-smokers. NGS alone was sufficient for molecular characterisation in 19.4% of cases; in 75.8%, an additional single molecular test was required. KRASm subtypes were distributed as follows: G12C (33.8%), G12V (25.8%), G12D (21%) and Q61H (6.5%). G12V was more prevalent in non-smokers (60%). Co-mutations were detected in 24.2% of patients, with PIK3CA being the most frequent. PD-L1 expression >50% was observed in 19.4% of patients. No significant associations were identified between KRAS subtypes and PD-L1 expression levels or co-mutations.

Significant differences in the clinical stage were noted across KRASm subtypes. Early-stage disease accounted for 24.19% of KRASm cases, with G12D observed in 40% of these patients. However, G12C and G12V subtypes were more frequently associated with metastatic disease (*p* = 0.004). While differences in median overall survival were observed across KRASm subtypes, they were not statistically significant (*p* = 0.5). The presence of co-mutations and high PD-L1 expression was suggested to be associated with a worse prognosis, without reaching statistical significance (*p* = 0.4 and *p* = 0.06, respectively).

**Conclusion:**

This study underscores the importance of assessing KRAS status and subtypes in NSCLC, particularly in early-stage disease, due to their association with metastatic risk. This could have relevance in treatment strategies and subsequent monitoring, which could necessarily be closer in higher risk patients. Moreover, while PD-L1 status shows potential as a prognostic factor in KRASm patients, further research is needed to confirm this relationship.

## Introduction

Rat sarcoma virus (RAS) is a well-known oncogene encoding small G proteins with intrinsic GTPase activity. This activity inactivates the protein and activates downstream effectors involved in multiple pathways, playing a critical role in cellular processes such as survival, division and differentiation. The RAS family includes three GTPase isoforms: Kirsten rat sarcoma virus (KRAS), neuroblastoma RAS viral oncogene homolog (NRAS) and Harvey rat sarcoma virus (HRAS). All three operate within the RAS-RAF-MEK-ERK (MAPK) pathway, which is activated by growth factors, cytokines, immunological receptors, integrins and chemokine receptors [1, 2].

Deregulation of the RAS oncogene disrupts survival, division and proliferation processes, leading to carcinogenesis. These disruptions may involve impaired receptor signalling, imbalances in BCL-2 family proteins regulating apoptosis, increased expression of inhibitors of apoptosis proteins, reduced caspase expression and mutations in tumour suppressor genes [2, 3]. Alterations in RAS oncogenes, particularly at codons 12, 13 or 61, are implicated in the development of various tumours. These mutations are present in up to 20% of cancers, with RAS mutation or amplification being the most common genetic alteration in human tumours [4–8].

Within the RAS family, KRAS is the most frequently mutated isoform, observed in approximately 17% of solid tumours, including 90% of pancreatic cancers, 50% of colorectal cancers and 25% of lung cancers. In non-small cell lung cancer (NSCLC), KRAS mutations are found in about 80% of lung adenocarcinomas [9, 10]. Alterations in HRAS and NRAS are less common and are mainly associated with melanoma, thyroid cancer (NRAS), bladder cancer, non-melanoma skin cancers and head and neck cancers (HRAS) [11].

Recent studies have identified distinct KRAS mutation subtypes, with KRAS G12C being the most prevalent in NSCLC, followed by G12V and G12D [12–15]. These subtypes have unique roles in carcinogenesis and drug response. For example, KRAS G12C and G12V increase RAS-related protein signalling but reduce PI3K/AKT signalling compared to other KRAS mutations or wild-type cell lines, while KRAS G12D preferentially activates the PI3K/AKT pathway [14, 16–20].

KRAS mutations are generally exclusive of other actionable driver mutations, such as EGFR, ROS1, ALK, MET, RET, BRAF, HER2 and NTRK. However, co-mutations in tumour suppressor genes such as STK11, TP53 or CDKN2A/CDKN2B are frequently observed in lung cancer. The presence of KRAS is significant not only for ruling out other targeted mutations but also for its potential as a therapeutic target. Recently, drugs targeting KRAS G12C, such as Sotorasib and Adagrasib, have been developed. These irreversible and selective inhibitors have shown clinical benefits in previously treated NSCLC patients. Sotorasib is European Medicines Agency and Food and Drug Administration approved as a second-line treatment, with ongoing studies evaluating its role in first-line therapy [21, 22].

The impact on the prognosis of the different pathological and clinical characteristics that accompany the KRAS mutation and their influence on it remains controversial. Different KRAS subtypes, co-mutations and PD-L1 expression levels may influence prognosis, treatment response and outcomes [23–35]. Most studies on KRAS mutations focus on metastatic disease, with limited data on the localised disease. Thus, information on KRAS is, in many cases, incomplete, since it is not clear whether these characteristics have an impact on the stage and aggressiveness of the disease. This document aims to evaluate the predictive role of KRAS subtypes by examining their relationship with clinical characteristics, disease stage and the impact of co-mutations and PD-L1 expression on patient survival.

## Methods

This study included 123 patients with various stages of lung cancer treated at our institution between January 2019 and December 2021. All patients underwent molecular analysis using next generation sequencing (NGS) with the AmpliSeq for Illumina Focus Panel. This targeted resequencing assay analyses 52 genes associated with carcinogenesis and tumour development, including ABL1, AKT1, AKT3, ALK, AR, AXL, BRAF, CCND1, CDK4, CDK6, CTNNB1, DDR2, EGFR, ERBB2, ERBB3, ERBB4, ERG, ESR1, ETV1, ETV4, ETV5, FGFR1, FGFR2, FGFR3, FGFR4, GNA11, GNAQ, HRAS, IDH1, IDH2, JAK1, JAK2, JAK3, KIT, KRAS, MAP2K1, MAP2K2, MET, MTOR, MYC, MYCN, NRAS, NTRK1, NTRK2, NTRK3, PDGFRA, PIK3CA, PPARG, RAF1, RET, ROS1 and SMO. The gene selection was based on published literature, established clinical guidelines such as those from the Association for Molecular Pathology, College of American Pathologists and European Society for Medical Oncology, as well as ongoing clinical trials.

The NGS platform analysed DNA and RNA simultaneously, detecting single nucleotide polymorphisms, gene fusions, somatic variants, insertions-deletions (indels) and copy number variants. In cases where NGS analysis was insufficient or defective, additional testing methods such as immunohistochemistry, fluorescence *in situ* hybridisation or real-time polymerase chain reaction were utilised. PD-L1 expression was assessed for all patients using the SP263 antibody.

**Figure 1.**
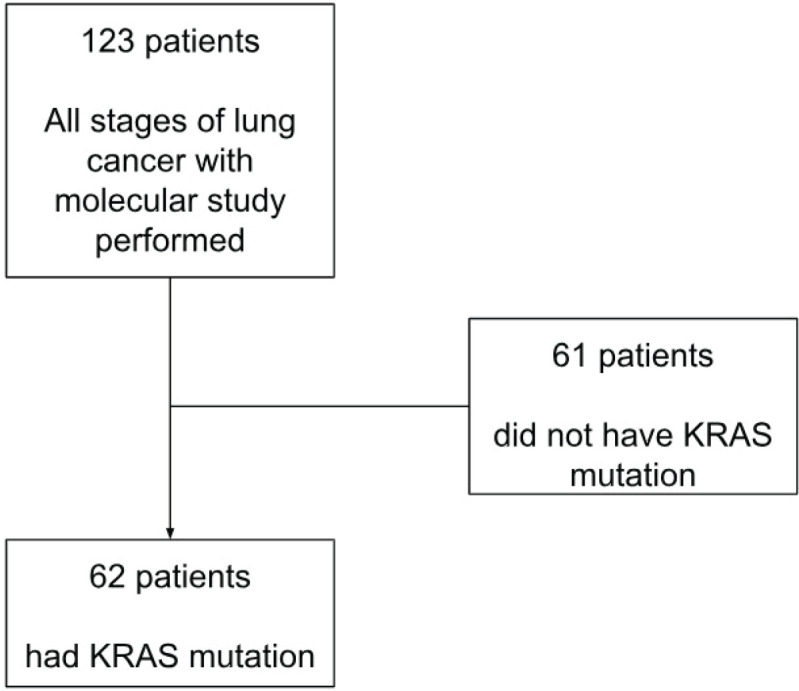
Patients flowchart. Of the 123 patients, 62 had mutations in the KRAS oncogene and were selected as the final cohort.

Out of the initial cohort of 123 patients, 62 were identified as having KRAS mutations and formed the final study group (Figure 1). Data collected for these patients encompassed molecular characteristics, including the specific KRAS mutation subtype, the presence of co-mutations and the percentage of PD-L1 expression. Clinical characteristics, such as sex, ethnicity, age at diagnosis, Performance Status (ECOG) and smoking history, were also recorded, along with disease-specific features such as histology type and stage.

To assess the relationship between KRAS subtypes and various molecular, clinical and disease characteristics, chi-square and logistic regression tests were performed to compare mean differences among patient groups. Statistical significance was defined as a *p* value of less than 0.05, using a two-sided type I error. All statistical analyses were conducted using the SPSS Package version 25.

Additionally, the study evaluated the influence of KRAS subtypes, co-mutations and PD-L1 expression on patient survival. Kaplan–Meier analysis was employed to assess survival outcomes, exploring the potential prognostic significance of these factors.

## Results

The study population comprised 62 patients with KRAS mutations, selected from an initial cohort of 123 lung cancer patients treated at the institution between January 2019 and December 2021. The median age of the patients was 67 years, ranging from 49 to 92 years. The majority were male, accounting for 79% of the cohort and nearly all were Caucasian (98.4%). Most patients had an ECOG Performance Status score of 0 (24.2%) or 1 (50%) at the time of diagnosis. A significant proportion were current or former smokers, representing 91.9% of the group.

Histologically, 87.1% of the tumours were adenocarcinomas. Regarding disease stage at diagnosis, the cohort included patients across all clinical stages: 24.2% were in stages I or II, 11.3% in stage III and 64.5% in stage IV. Molecular analysis was performed using NGS alone in 24.2% of cases, while 75.8% required a combination of NGS and an additional single test. PD-L1 status was determined for nearly all patients, with data missing for only one individual (Table 1).

### KRAS subtypes and clinical-molecular characterisation

**Figure 2.**
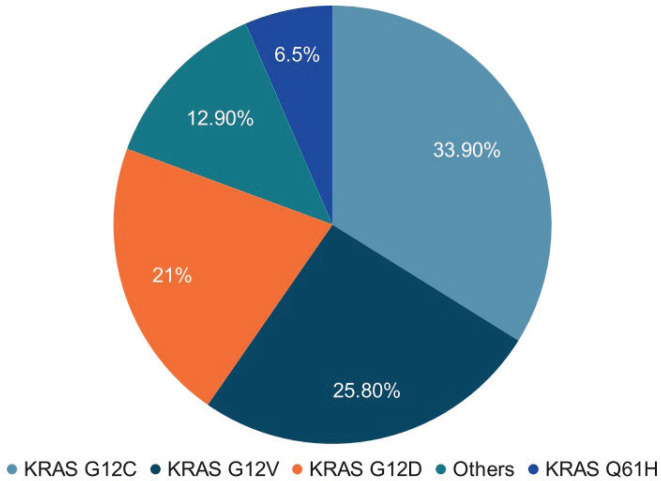
KRAS subtypes. In our cohort of 62 lung cancer patients with KRAS mutations, the most frequent subtype was G12C (33.8%), followed by G12V (25.8%), G12D (21%) and Q61H (6.5%). Eight patients (12.8%) had other KRAS mutation subtypes.

In this cohort of 62 lung cancer patients with KRAS mutations, the most common subtype was KRAS G12C, detected in 33.8% of cases. This was followed by KRAS G12V (25.8%), KRAS G12D (21%) and KRAS Q61H (6.5%), while 12.8% of patients had other less frequent KRAS mutation subtypes (Figure 2).

Among patients with the KRAS G12C mutation, all were smokers, and adenocarcinoma histology was observed in 95.2% of cases. Most of these patients (76.2%) were diagnosed at stage IV, with 76.2% showing negative PD-L1 expression. The most frequent co-mutation in this subtype was a RET rearrangement.

The KRAS G12V subtype, the second most frequent, showed a higher percentage of non-smokers (18%) compared to KRAS G12C. While adenocarcinoma histology remained predominant (75%), 81.3% of these patients were diagnosed at stage IV. PD-L1 positivity was seen in 43% of cases. Co-mutations were detected in this group, but none showed a notably higher prevalence (Table 2).

Patients with the KRAS G12D subtype, like those with KRAS G12V, included a larger proportion of non-smokers. Interestingly, this subtype was associated with earlier disease stages, with 53.9% of patients diagnosed at stages I, II or III, compared to KRAS G12C (23.8%) and KRAS G12V (18.9%). A majority (53.8%) of KRAS G12D patients exhibited negative PD-L1 expression. This subtype also demonstrated the lowest rate of co-mutations, with only one patient presenting a co-mutation involving KRAS itself (Table 2).

KRAS Q61H was the least frequent subtype, identified in only four patients. Half of these cases were diagnosed at early stages, and PD-L1 expression was observed in 50% (Table 2).

**Figure 3.**
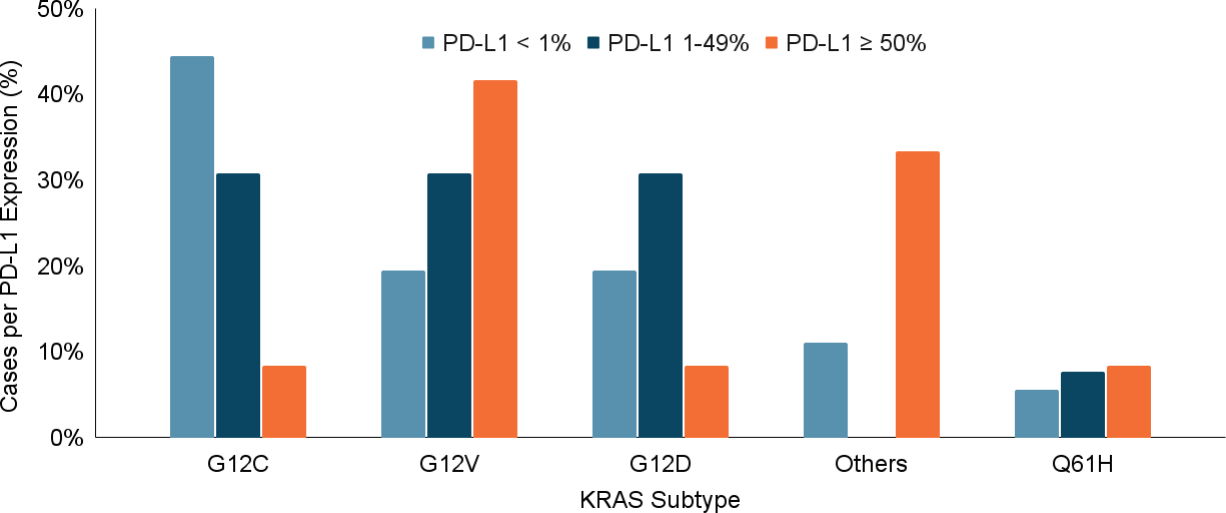
(a–d): Relationship between KRAS mutation subtype and clinical stage, presence of metástasis, PD-L1 expression and presence of co-mutations. A statistically significant relationship was identified between the different clinical stages and the KRAS subtyes, with the KRAS G12D subtype being more frequent in patients with early-stage disease (p = 0.04) (a). In the same way, the KRAS subtype was related to the presence of metástasis, presenting a higher incidence of metastasis in those patients with KRAS G12C and G12V subtype mutations (p = 0.004) (b). On the contrary, the KRAS subtype was not significantly related to the percentage of PD-L1 expression (p = 0.16) (c) nor to the presence or absence of co-mutations (p = 0.26) (d).

Statistical analysis did not reveal significant differences in sex (*p* = 0.9), ethnic origin (*p* = 0.7), smoking habit (*p* = 0.4) or histology (*p* = 0.6) among the various KRAS mutation subtypes. However, a statistically significant relationship was observed between the clinical stage and KRAS subtypes. KRAS G12D was more common in early-stage disease (*p* = 0.04), while KRAS G12C and KRAS G12V were associated with higher rates of metastatic disease (*p* = 0.004). There was no significant association between the KRAS subtype and PD-L1 expression levels (*p* = 0.16) or the presence of co-mutations (*p* = 0.26) (Figure 3a–d).

### KRAS subtypes and overall survival

The analysis revealed no significant differences in overall survival among patients based on the subtype of KRAS mutation (*p* = 0.6). Similarly, survival outcomes did not differ significantly between patients with or without co-mutations, with median survival times of 11 and 8 months, respectively (*p* = 0.6).

**Figure 4.**
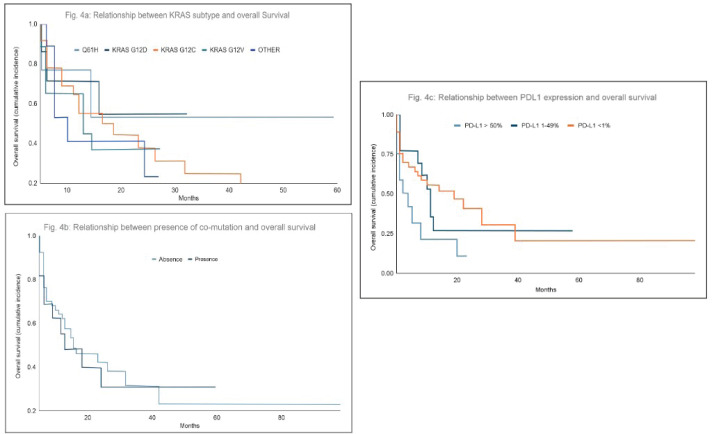
(a–c): Relationship between KRAS mutation subtype, PD-L1 expression or presence of co-mutations and overall survival. There were no significant differences in overall survival based on the KRAS mutation subtype (p = 0.6) (a) and co-mutations (p = 0.6) (b). In terms of PD-L1 expression, although statistical significance was not reached, patients with negative PD-L1 expression tended to have greater survival than those with PD-L1 expression >50% (p = 0.068) (c).

Regarding PD-L1 expression, although statistical significance was not achieved, a trend was observed suggesting that patients with negative PD-L1 expression may have better survival outcomes compared to those with higher levels of PD-L1 expression. Specifically, median survival was 19 months for patients with PD-L1 <1%, 11 months for those with PD-L1 expression between 1%–49% and 8 months for patients with PD-L1 >50% (*p* = 0.068) (Figure 4a–c).

## Discussion

Lung cancer remains the most common cancer globally and the leading cause of cancer-related deaths.

Tobacco is a major carcinogen linked to lung cancer, but other genetic triggers, particularly mutations in KRAS, EGFR, ALK, BRAF, RET, MET and ROS1, are also central to its development. Among these, KRAS mutations are the most frequent, present in 80% of lung cancer cases, particularly in adenocarcinoma subtypes and are largely related to smoking. The most common KRAS mutations are G12C, G12V and G12D, with emerging evidence indicating that KRAS mutations negatively impact prognosis, contributing to poorer outcomes and shorter survival following first-line treatment. With the availability of new therapies like Sotorasib, which targets KRAS G12C mutations in NSCLC, identifying factors that help pinpoint patients more likely to harbour these mutations is crucial [36, 56].

Our epidemiological data support previous research, showing strong associations between KRAS mutations, smoking and adenocarcinoma histology, with 91.9% of patients being smokers and 87.1% having adenocarcinoma. The study also found a predominance of male patients (79%). The most common KRAS mutation was G12C, followed by G12V and G12D, which is consistent with earlier studies. However, no statistically significant links were found between KRAS subtype mutations and factors such as sex, histology or smoking habits.

At the time of diagnosis, most patients were diagnosed at advanced stages, with 64.5% presenting with stage IV disease. This mirrors findings in other studies showing a higher frequency of advanced disease among patients with KRAS mutations. The study also identified significant differences in the frequency of metastasis and clinical stages across KRAS subtypes. KRAS G12C and G12V mutations were associated with higher rates of metastasis (76.2% and 81.3%, respectively) compared to the G12D subtype, which had a higher frequency of localised stages at diagnosis (53.9%). These findings suggest that different KRAS mutation subtypes may exhibit distinct behaviour, leading to varied outcomes. Patients with the KRAS G12D mutation, in particular, appeared to have better survival outcomes, with their survival not yet reached at the time of analysis.

Regarding PD-L1 expression, the study found that 19.4% of patients had a PD-L1 expression greater than 50%. Interestingly, the expression levels of PD-L1 varied by KRAS mutation subtype. Although not statistically significant, the data suggested that patients with KRAS G12V mutations had higher PD-L1 expression, whereas those with KRAS G12C mutations had higher rates of PD-L1 negativity. Previous studies suggest that KRAS mutations may create an immunologically active tumour microenvironment, potentially enhancing the effectiveness of immunotherapies. KRAS-mutant NSCLC patients tend to respond better to immunotherapy compared to KRAS wild-type patients, particularly when PD-L1 expression is elevated. Despite this, our study found that patients with PD-L1 expression above 50% had worse survival outcomes. This could be due to the fact that many of these patients had more aggressive KRAS subtypes like G12V, which are more often associated with advanced disease.

Although KRAS mutations are generally mutually exclusive with other oncogenic mutations, co-mutations in tumour suppressor genes such as STK11, TP53 or KEAP1 are frequently seen in KRAS-mutant tumours. These co-mutations are associated with treatment resistance in preclinical models and may contribute to a poor prognosis. STK11 mutations are particularly problematic, as they are linked to resistance to both immunotherapy and chemotherapy. TP53 mutations, which are among the most common genetic alterations in lung cancer, can promote resistance to chemotherapy agents and may also alter the tumour’s immune environment, reducing the effectiveness of immune checkpoint inhibitors. Furthermore, co-mutations in KEAP1, a gene involved in regulating the antioxidant response, are also commonly seen in KRAS-mutant tumours. KEAP1 mutations often result in the activation of the Nrf2 pathway, which can lead to the development of resistance to chemotherapeutic agents, particularly those that generate oxidative stress as a mode of action.

While it is interesting to use our panel to explore the frequency of mutations in lesser known genes or those closely related to KRAS, the study of KEAP1, STK11 and TP53 is noteworthy for their already-known role in these tumours. Identifying and understanding these co-mutations is essential for improving patient prognosis, as they could inform the development of combination therapies that target both the KRAS mutation and the associated tumour suppressor gene alterations.

The small sample size of our study restricts the generalisability of our findings and limits statistical power, especially when assessing the significance of KRAS mutation subtypes or co-mutations. Although trends were observed suggesting differences in aggressiveness and metastatic potential between KRAS G12C, G12V and G12D subtypes, the small sample size may have impacted our ability to detect truly significant differences between these subtypes.

Moreover, the heterogeneity of the patient population and the diversity of genetic and clinical subtypes make it even more challenging to detect precise patterns when the sample size is limited. Studies with larger sample sizes allow for greater precision in effect estimation, which is essential for establishing reliable relationships between KRAS mutations and survival outcomes. In this regard, larger studies are necessary not only to confirm the preliminary observations made but also to help identify mutation patterns and their clinical implications more robustly.

## Conclusion

In conclusion, our study underscores the importance of personalised treatment approaches in NSCLC, particularly for patients with KRAS mutations. The differing behaviours of KRAS mutation subtypes suggest that treatment strategies should be tailored based on the specific subtype of KRAS mutation, with the potential for more targeted therapies or immunotherapy. Moreover, these findings could have relevance in treatment strategies and subsequent monitoring, which could necessarily be closer in higher risk patients.

Future research should focus on larger cohort studies with increased statistical power and the inclusion of critical co-mutations such as STK11, TP53 and KEAP1, to better understand their role in treatment resistance and prognosis. Expanding molecular analyses to include a wider array of genetic alterations could significantly improve the management and outcomes of KRAS-mutant NSCLC.

## Statements of ethics

The study was carried out following Good Clinical Practices, in accordance with the Declaration of Helsinki (Fortaleza, Brazil, October 2013), and with the biomedical research law 14/2007, of July 3. This study included no clinical trials or animal experiments. All patients with this pathology have a molecular study within the healthcare practice, so this study did not involve extra procedures beyond the usual healthcare process. Therefore, the study has been granted an exemption from requiring written informed consent. The ethical approval was conceded by the CEIm committee (Research Ethics Committee of the Parc Tauli Hospital Center).

## Conflicts of interest

The authors have no conflicts of interest to declare.

## Funding

The authors declare that no funds, grants or other support were received during the preparation of this manuscript.

## Author contributions

Study conceptualisation and design: Carla Climent, Julia Giner, Laia Vilà.

Acquisition, analysis or interpretation of data: Carla Climent, Sandra Soriano, Julia Giner, M Carmen Blazquez, Ruben Carrera, Natalia Lopez, Marina Sierra, Pablo Cobo, Monica Fragio, Mireia Busquets, Ona Cano I Cano, Alicia Carrasco, Laia Vila.

Drafting of the manuscript: Carla Climent, Sandra Soriano, Julia Giner, Natalia Lopez, Marina Sierra, Pablo Cobo, Monica Fragio, Mireia Busquets, Ona Cano I Cano, Alicia Carrasco, Laia Vila.

Critical revision and final approval of the manuscript: Carla Climent, Julia Giner, Laia Vilà.

Administrative, technical or material support: Carla Climent, Julia Giner, M Carmen Blazquez, Ruben Carrera, M Angel Seguí, Laia Vila.

## Data availability statement

We were allowed to access the ONQOS database after registration on the official website. All data in this study are from the ONQOS database. Further inquiries can be directed to the corresponding author.

## Figures and Tables

**Table 1.** Baseline characteristics of patients

**Age (years)**	67 (49–92)
Sex	
°Men	49 (79%)
°Women	13 (21%)
Ethnic origin	
°Caucasian	61 (98.4%)
°Afrodescendants	1 (1.6%)
ECOG	
°0	15 (24.2%)
°1	31 (50%)
°2	9 (14.5%)
°3	7 (11.3%)
Smoking habit	
°Former/Current	57 (91.9%)
°Never	5 (8.1%)
Histological subtype	
°Adenocarcinoma	54 (87.1%)
°Squamous	1 (1.6%)
°Others	7 (11.3%)
Clinical stage	
°I-II	15 (24.2%)
°III	7 (11.3%)
°IV	40 (64.5%)
Molecular evaluation	
°NGS	12 (19.4%)
°NGS + Single test	47 (75.8%)
°Single test	3 (4.8%)
Evaluation PD-L1 expression	
°Yes	61 (98.5%)
°No	1 (1.6%)

**Table 2.** Patient characteristics.

	G12C (*n* = 21)	G12V (*n* = 16)	G12D (*n* = 13)	Q61H (*n* = 4)	Others (*n* = 8)
Age (years)	66.5	69.8	63.6	71.7	65.6
Sex					
°Men	17 (81%)	12 (75%)	11 (84.6%)	3 (75%)	6 (75%)
°Women	4 (19%)	4 (25%)	2 (15.4%)	1 (25%)	2 (25%)
Ethnic origin					
°White	20 (95.2%)	16 (100%)	13 (100%)	4 (100%)	8 (100%)
°Black	1 (4.8%)	-	-	-	-
ECOG					
°0	4 (19%)	2 (12.5%)	6 (46.2%)	-	3 (37.5%)
°1	11 (52.4%)	11 (68.8%)	4 (30.8%)	2 (50%)	3 (37.5%)
°2	1 (4.8%)	2 (12.5%)	3 (23.1%)	1 (25%)	2 (25%)
°3	5 (23.8%)	1 (6.3%)	0	1 (25%)	-
Smoking habit					
°Yes	21 (100%)	13 (81.3%)	11 (84.6%)	4 (100%)	8 (100%)
°No	-	3 (18%)	2 (15.4%)	-	-
Histological subtype					
°Adenocarcinoma	20 (95.2%)	12 (75%)	11 (84.6%)	3 (75%)	8 (100%)
°Squamous	-	1 (6.3%)	-	-	-
°Others	1 (4.8%)	3 (18.8%)	2 (15.4%)	1 (25%)	-
Clinical stage					
°I-II	3 (14.3%)	2 (12.6%)	6 (46.2%)	2 (50%)	2 (25%)
°III	2 (9.5%)	1 (6.3%)	1 (7.7%)	1 (25%)	2 (25%)
°IV	16 (76.2%)	13 (81.3%)	6 (46.2%)	1 (25%)	4 (50%)
PD-L1 expression ≥1					
°Yes	5 (23.8%)	7 (43.8%)	5 (38.5%)	2 (50%)	4 (50%)
°No	16 (76.2%)	9 (56.3%)	7 (53.8%)	2 (50%)	4 (50%)
Co-mutation					
°Yes	5 (23.8%)	6 (37.5%)	1 (7.7%)	2 (50%)	2 (25%)
°No	16 (76.2%)	10 (62.5%)	12 (92.3%)	2 (50%)	6 (75%)
	1 BRAF mutation not V600E 2 RET re arrangements 1 FGFR2 mutation 1 PI3KCA mutation	1 EGFR mutation 1 PDGFRA mutation 1 PIK3CA mutation 1 JAK2 mutation 1 C-KIT mutation 1 NRAS mutation	1 KRAS co-mutation	1 ERBB2 + MAP2KI co-mutation 1 mTOR mutation	
